# Intergenerational Ethical Issues and Communication Related to High-Level Nuclear Waste Repositories

**DOI:** 10.1007/s40572-019-00257-1

**Published:** 2019-11-12

**Authors:** Martin Tondel, Lena Lindahl

**Affiliations:** 1 Occupational and Environmental Medicine, Department of Medical Sciences, Uppsala University, SE-751 85 Uppsala, Sweden; 2 Occupational and Environmental Medicine, Uppsala University Hospital, Uppsala, Sweden; 3 Connect Japan LLC, 1-47-8 Kashima, Chuodai, Iwaki City, Fukushima Prefecture, 970-8043 Japan

**Keywords:** Björketorp, Runestone, Tomb, Tsunami, Radioactive, KBS

## Abstract

**Purpose of Review:**

The nuclear power industry started in the 1950s and has now reached a phase of disposing high-level nuclear waste. Since the 1980s, the United Nations has developed a concept of sustainable development and governments have accordingly made ethical commitments to take responsibility towards future generations. The purpose of this review is to examine ethical dilemmas related to high-level nuclear waste disposal in a long-term perspective including potential access to the waste in the future. The time span considered here is 100,000 years based on current experts’ assessment of the radiological toxicity of the waste.

**Recent Findings:**

In this review, we take into account findings on ethical issues related to the disposal of high-level nuclear waste put forward by the Radioactive Waste Management Committee (RWMC), the International Commission on Radiological Protection (ICRP), nuclear waste management companies (SKB in Sweden and Posiva Oy in Finland), and several researchers. Some historical examples are presented for potential guidance on methods of communication into the future.

**Summary:**

According to the sustainable development ethical principle, adopted by the United Nations, we conclude that governments with nuclear energy have committed themselves to protect future generations from harm related to high-level nuclear waste. This commitment involves the necessity to convey information together with the nuclear waste. Our paper examines disposal options chosen by Sweden and Finland, as well as some contemporary and historical efforts to design messages towards the future. We conclude that the international community still needs to find methods to communicate in an intelligible way over long periods of time.

## Background

In 1987, a definition of sustainable development was launched taking the rights of future generations into account. Sustainable development was defined as development that meets the needs of the present without compromising the ability of future generations to meet their own needs [1]. This concept of sustainable development has now been globally accepted and is integrated in the 2030 Agenda for Sustainable Development adopted by the United Nations General Assembly in 2015 [2]. The nuclear power industry started in the 1950s and has now reached a phase of disposing high-level nuclear waste. The sustainable development ethical principle has made it a task of national governments to ensure that their nuclear industries use methods for disposal of high-level nuclear waste that respect this principle as to prevent harm for future generations. One interpretation of sustainable development is that current generations are obliged to communicate both risks and possibilities of high-level nuclear waste repositories, to future generations. Our paper looks into the methods for communicating critical information over time periods spanning thousands of years.

Many countries have high-level nuclear waste in interim storages and are considering different options on how to safely dispose the waste. A newly released report discusses retrievability, but also the concept of a Set of Essential Records (SER) as an important component of a Records, Knowledge, and Memory (RK&M) preservation strategy. The SER is designed to be a compilation of actual records, selected because they would be required for future generations to understand the repository system and its performance, as well as to make informed decisions [3•]. However, that report has a limited time frame as it describes documentation only relying on paper material and only in English language and therefore gives few answers about how to communicate in a time perspective of several thousand years.

Sweden and Finland are among few countries that have already decided locations and methods for high-level nuclear waste repositories. Both countries have chosen the KBS-3 (nuclear fuel safety-3) method for final disposal. It is called KBS-3 because it uses three protective barriers: copper canisters, bentonite clay, and bedrock. The three barriers are expected to prevent radionuclides from reaching the biosphere for 100,000 years [4]. Swedish law requires companies that operate nuclear power plants in Sweden to pay for all the costs associated with the final disposal of nuclear waste (the polluter pays principle). Nuclear power plant operators own the Swedish Nuclear Fuel and Waste Management Company SKB which is responsible for finding a safe method for the final disposal of the spent nuclear fuel. In Finland, a similar company, Posiva Oy, has a corresponding assignment to build a safe disposal facility for the spent nuclear fuel with minimal risk to people and the environment. The KBS-3 method means that the nuclear waste will not be reprocessed, but instead buried at a depth of 500 m in the ground. Sweden and Finland have decided not to reprocess the spent nuclear fuel. One reason was an early ethical consideration because reprocessing could make the material available for nuclear weapons production. The waste repositories will be filled to full capacity with spent fuel from all current domestic nuclear power plants in the respective countries, not allowing import of nuclear waste. An estimated 6000 canisters will be deposited during the operating phase of the Swedish nuclear waste repository. It will then be backfilled and sealed with a final closure year in 2090. Finland plans to seal their repository in 2120. The intention of sealing is to prevent future access to the waste. In the case of SKB, the company will cease to exist after sealing the repository and the responsibility for future generations will then be taken over by the Swedish government. SKB is exploring communication options to fulfill the ethical obligation of warning future generations [4]. To see what can be learned about how to convey risks and messages into the future, this review looks at some historical examples of intergenerational communication attempts with similar intentions.

## Ethical Issues

There are two main issues of fairness between generations. The first is the responsibilities of the current generation towards future generations (intergenerational equity). The second is about how burdens and rights of decision-making are shared between people in the contemporary society (intragenerational equity) [5••, 6]. Our paper focuses on intergenerational equity as expressed in the sustainable development ethical principle.

Regarding the intergenerational equity, there is one basic question to consider. Should the final disposal site be sealed with the intention that future generations shall never access this waste (non-retrievability), or should future generations be able to retrieve the waste and process it with new technologies for the benefit of society (retrievability)? Does the current generation have an ethical right to make potentially useful metals and radionuclides, extracted with great effort and expense, unavailable for future generations? Who will bear financial burdens and health risks in the long term after sealing a repository? How can contemporary societies guarantee that knowledge about risks and possibilities is passed on to future generations to prevent harm regardless of retrievability?

## Intergenerational Equity

The ntergenerational equity concept has been adopted in the generational goal and the 16 environmental objectives decided by the Swedish Parliament in 1999. The generational goal is intended to guide environmental policy and action at every level of society: “The overall goal of Swedish environmental policy is to hand over to the next generation a society in which the major environmental problems in Sweden have been solved, without increasing environmental and health problems outside Sweden’s borders” [7].

The intention of adopting a generational goal is to provide basic conditions to solve environmental problems within one generation, i.e., by the year 2025. The time perspective set by this generational goal is interesting compared to the time frame for the nuclear waste repository. The Swedish Parliament is only able to plan for a few decades, while the radioactive waste must be safely stored for 100,000 years. This long-term perspective is a challenge to society in several ways especially because the current generation needs to guess what future society will look like and how much responsibility people in the future will take for their successors. For example, for how long can a state or nation be expected to exist? It is unlikely that national borders will remain forever, so who will be responsible for the nuclear waste located in areas today regarded as Swedish or Finnish?

If the current generation fails to keep the highly radioactive waste isolated from the environment and people for a long period of time and it causes severe damage to future generations, how will people in the far future judge them? If future generations understand warnings and take archives into account, will they understand that the predecessors did their best to protect them?

## Retrievability

If future generations were given access to the high-level nuclear waste (retrievability), it would give them a flexibility to monitor, repair, and replace leaking containers, and perhaps in a far future find a safer method to store the waste. Retrievability buys time until current technology has developed, and better decisions can be made about storing, moving, reprocessing, or permanently disposing the waste [8]. Waste disposal in a non-retrievable repository might, in a worst-case scenario, result in groundwater contamination that needs to be mitigated without access to the leaking containers [9]. Nevertheless, both Sweden and Finland have chosen a final repository solution with non-retrievability. The countries have come to this conclusion, relying on present knowledge, that the safest solution for future generations is non-retrievability in a deep burial site in order to avoid all possible harm to humans and the environment. A passively safe disposal, just waiting for the radioactivity to naturally decay, has the advantage of not being dependent on a highly skilled civilization in the future.

The non-retrievable repository is a strategy that encompasses intergenerational equity but on the other hand, it prevents future generations from taking advantage of the valuable resources in the waste deposit such as metals and fissile materials. This mindset is characterized by doubt that future generations will take responsibility for the inherited waste. Also, the risk assessment is based on present knowledge of the nuclear waste and limited imagination about how society will be organized in 100 years, even less so in 1000, 10,000, or 100,000 years. Could a future society with better knowledge and technology handle the waste with better wisdom than can be imagined today? Although extremely difficult to access, future societies might nevertheless consider the repository as a tempting mining site.

## Knowledge Transfer

In the film “Into Eternity” about the final repository in Onkalo, Finland, a question was asked about how warning texts for future generations should be designed to discourage people from opening the sealed Onkalo [10]? When it is difficult to imagine a time perspective of 100,000 years, a look into the past can be informative. Modern man (*Homo sapiens*) emerged about 300,000 years ago and the Neanderthals (*homo neanderthalensis*) died out about 30,000 years ago [11]. The famous cave paintings in Altamira, Spain, are only 14,000 years old and the first written language was created in Mesopotamia about 3100 BC [12, 13]. Is it ethical, or perhaps unethical, to design warning texts when we have no idea how the texts/symbols will be interpreted after thousands of years? The Radioactive Waste Management Committee (RWMC) and the International Commission on Radiological Protection (ICRP) conclude that the best way to minimize damage to future generations is to backfill and seal a final repository and to retain all relevant information in an archive of technical information about the waste and how it is stored [5••, 6, 14••]. The use of warning signs or markers to inform future generations on the existence of the archives/repositories may be considered [10]. How should such markers be designed? Would it be more ethically sound to leave NO markers or records at all, in the hope that the waste will never be found? How far into the future do current generations have a responsibility to pass on information about the repository? Should a mechanism be built for each generation to update the warning marks, and edit the language and symbols to ensure the message will be properly understood? If so, how will this be accomplished for thousands of years to come? We cannot fully interpret the cave paintings in Altamira, that are only 14,000 years old, so how can we invent a method to communicate over a much longer timespan?

## Historical Examples of Warnings

An ancient example of an effort to communicate risk over generations can be found in Egypt. The archeologist Zahi Hawass describes verses found in entrances to tombs from the Old Kingdom having the sole purpose of frightening and discouraging people so that they will not enter and disturb the souls of those buried there. One such inscription says “cursed be those that disturb the rest of the pharaoh. They that shall break the seal of this tomb shall meet death by a disease that no doctor can diagnose.” Hawass has also excavated tombs of the pyramid builders at Giza and then encountered this curse: “O all people who enter this tomb, who will make evil against this tomb and destroy it, may the crocodile be against them on water, and snakes against them on land. May the hippopotamus be against them on water, the scorpion against them on land” [15]. These warnings have apparently been ignored by both tomb raiders and archeologists.

**Fig. 1:**
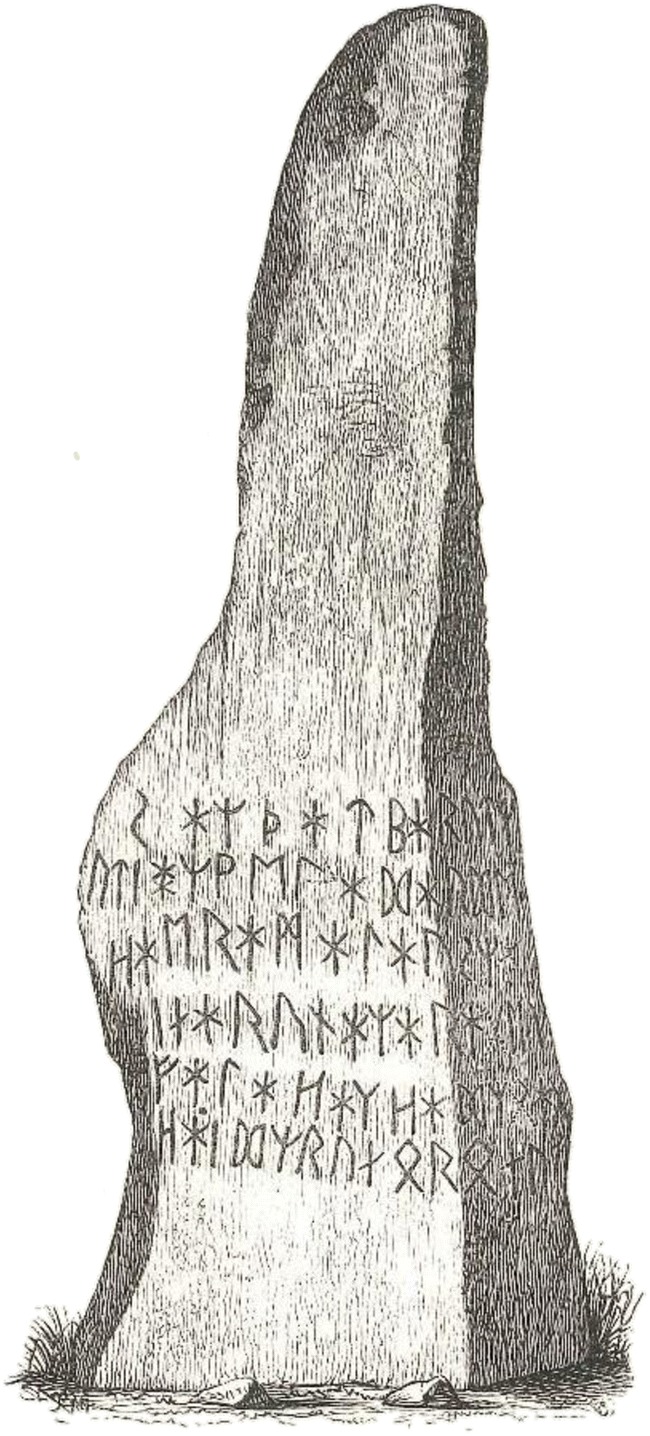
The Björketorp stone (height 3.5 m) [17]. The rune inscription has been deciphered, but several interpretations exist.

Two examples, more recent in geological terms, are Björketorpsstenen, a Swedish runestone, and tsunami stones in Japan. The first example is the inscription on the Björketorp stone dated 600–700 AD (Fig. 1). The exact interpretation of the inscription has been debated, but it is obviously intended to protect a grave. The inscription is magical in nature and one possible interpretation is: “I have here the secret meaning of powerful runes. He who destroys the monument will forever be tormented by evil witchcraft. He shall die a treacherous death. I prophesy ruin” [16]. Like the Egyptian curse, this Swedish curse has also the intention to protect against intruders. Again, this has not deterred, but rather encouraged, archeological excavations in hope of finding rich treasures.

**Fig. 2:**
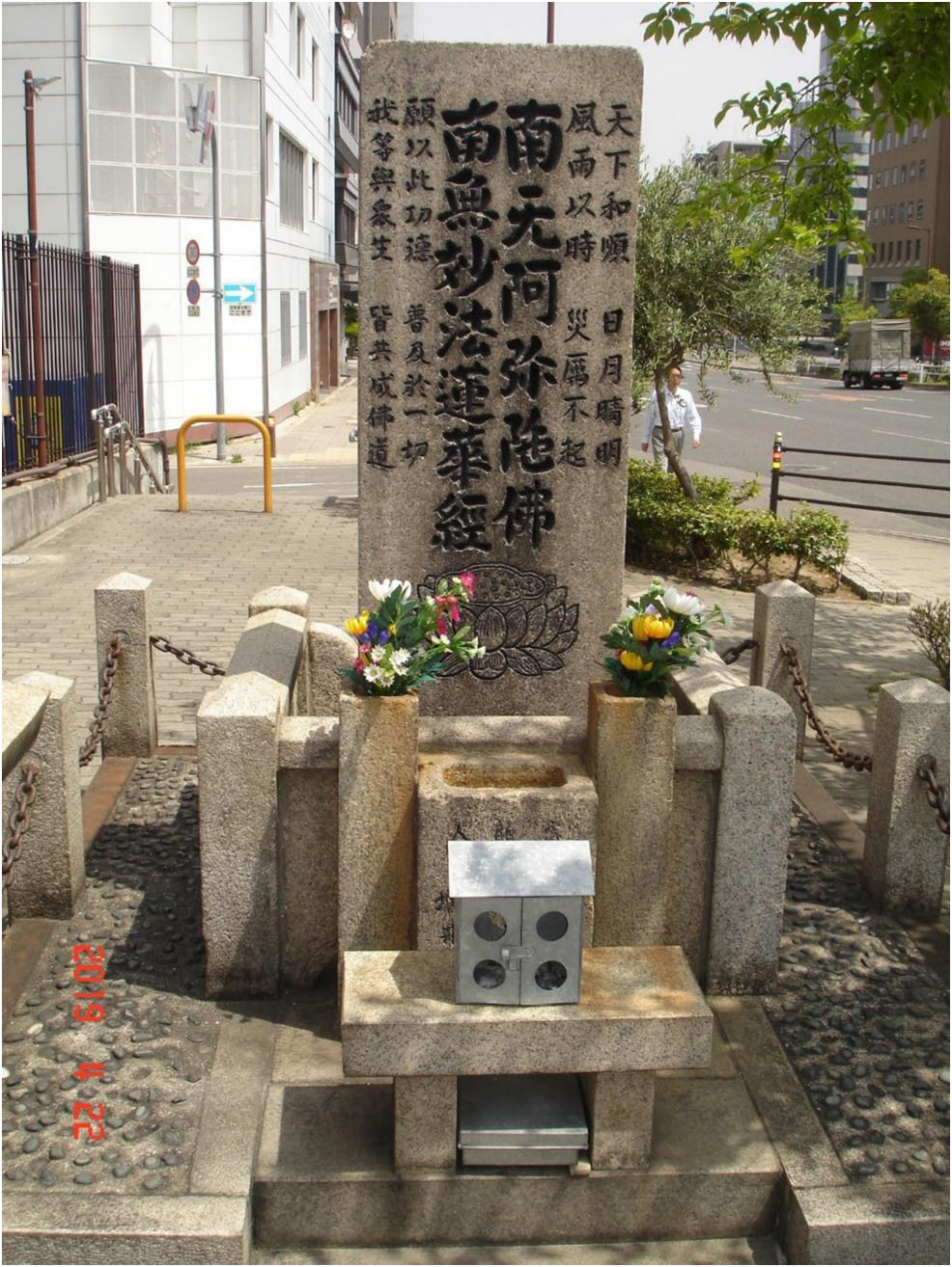
The Ansei Otsunami stone, Osaka. Warning text is on the back side. The inscription on this stone can be read and easily translated to modern Japanese language, but is unintelligible to contemporary people who do not know Japanese [19]. Photo: Martin Tondel

The second example is the Japanese tsunami stones intending to pass on memories and warnings to protect people and property from tsunami damage. All earthquakes and subsequent tsunamis since the great Jogan tsunami in 869 AD have been given their own names and hundreds of memorial tsunami stones have been placed along the coast of Japan. The Aneyoshi stone is of unknown age and carries the inscription: “High dwellings ensure the peace and happiness of our descendants. Remember the calamity of the great tsunami. Do not build any homes below this point.” Another tsunami stone in Kesennuma reads: “If earthquake comes, beware of tsunami” and “choose life over your possessions and valuables” [18•]. The Ansei Otsunami stone in Osaka was raised in 1855, a year after the large tsunami the year before (Fig. 2). The text calls upon people to escape to higher ground as soon as they experience an earthquake, a warning that a tsunami may hit the coast. The stone further tells people to sometimes fill in the inscriptions with black ink so that the text can be read and remembered. With some exceptions, most of these warning stones have been ignored and homes built near the shore. Some stones have been washed away by later and higher tsunamis and thereby the messages to future generations have been lost and efforts been in vain. Thus, previous generations in Japan did their best to fulfill their ethical responsibility to provide future generations with warnings, but often failed because large tsunamis are rare, people have short memories, and other reasons.

Older warnings than mentioned here might exist or may have been lost in history. Historical examples raise questions about how warning marks for a nuclear waste repository should be designed to last over millennia. Both in the West and the East, stone has been recognized as the most durable material to pass on messages to the next generation. As proven, stone has survived the passage of time in many cases. In Japan, another robust material has been suggested recently, silicon carbide, resistant far beyond stone, metal, or paper [20].

**Fig. 3:**
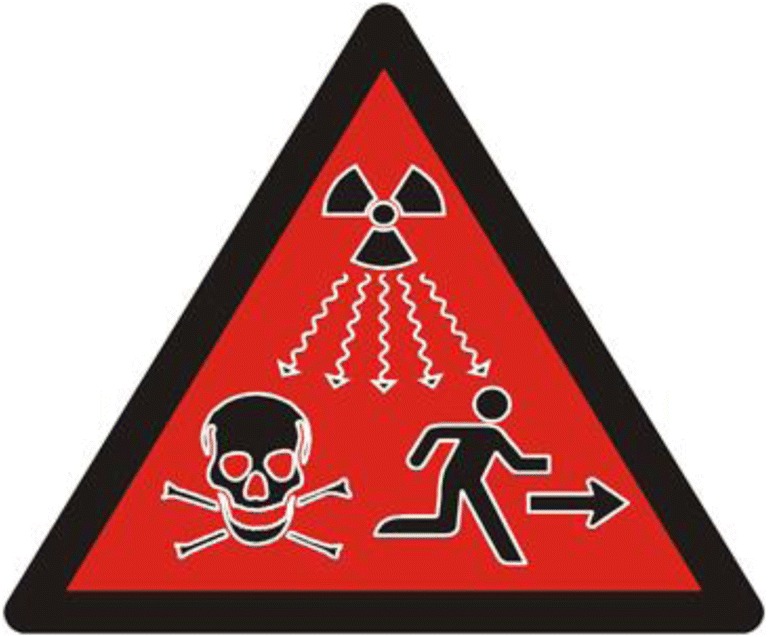
The IAEA launched a new non-verbal warning symbol in 2007 [21]

These Swedish and Japanese examples of rather recent markings, intended as eternal messages, have been ineffective. What will be the fate of markings with a message meant to be respected for many 10,000 years? The film “Into Eternity” suggests that messages conveying a feeling can be more successful, but can we be sure that our ways of expressing feelings are consistent over time [10]? Figure 3, launched by International Atomic Energy Agency (IAEA), has the intention of describing danger. Is the interpretation of this sign consistent in all cultures, over time, and in all situations?

SKB has made a proposal for an action plan that ultimately has the purpose to ensure that the information on the final repository for spent nuclear fuel can be preserved into the future. So far, the action plan is concrete for only the next 5 years, but with an aim to be updated. The purpose of the proposed action plan by SKB is to present ideas on concrete measures and guidelines in the short- and long-term regarding the work on information preservation [22].

## Summary and Conclusions

A concept of sustainable development has gained international acceptance through agreements in the United Nations and includes responsibility of current generations towards future generations. This can be interpreted to mean that governments with nuclear energy have committed themselves to protect future generations from harm related to high-level nuclear waste. Sustainability can also be interpreted as the right of future generations to access the waste and handle it according to their ethical principles. The sustainability commitment involves the necessity to convey information including the concept of sustainable development together with the nuclear waste. This conclusion applies to both retrievable and non-retrievable repositories.

In our paper, we have examined the non-retrievable option, a few historical examples of warnings, and disposal methods for high-level nuclear waste chosen by two countries. We conclude that the international community still needs to find methods to communicate in an intelligible way over long periods of time. Based on what we know today, non-retrievable nuclear waste repositories will become mausoleums for nuclear waste with ambitions to communicate messages over longer periods of time than ever before.
